# Valproic acid promotes mitochondrial dysfunction in primary human hepatocytes in vitro; impact of C/EBPα-controlled gene expression

**DOI:** 10.1007/s00204-020-02835-x

**Published:** 2020-07-04

**Authors:** F. Caiment, J. Wolters, E. Smit, Y. Schrooders, J. Kleinjans, T. van den Beucken

**Affiliations:** Maastricht University, Maastricht, The Netherlands

**Keywords:** Steatosis, Valproic acid, RNA interference, Lentiviral shRNA, Transcription factors, Ccaat/enhancer-binding protein alpha (C/EBPα), Oxygen consumption, Primary human hepatocytes

## Abstract

Valproic acid (VPA) is a frequently prescribed anti-epileptic drug which is known to cause liver toxicity and steatosis through mitochondrial dysfunction. Nevertheless the mechanisms underlying these adverse effects are incompletely understood. In this study, we determined the effect of relatively short (3 h) or prolonged (72 h) exposure to VPA on mitochondrial function in primary human hepatocytes (PHHs). While 3 h VPA exposure did not affect oxygen consumption rates (OCRs) in PHHs, prolonged exposure (24–72 h) significantly reduced basal and maximal OCRs. Given that in particular prolonged VPA exposure is required to cause mitochondrial dysfunction, we investigated gene expression data after VPA exposure for 24, 48, 72 h and 72 h VPA followed by a 72 h washout period. We were able to reduce the comprehensive gene expression changes into a more comprehensible set of 18 TFs that were predicted to be persistently activated after 72 h of VPA exposure. Lentiviral knock-down of one of the candidate TFs, C/EBPα, partly rescued VPA-induced mitochondrial dysfunction. Furthermore, RNA-Seq analysis of shC/EBPα and shGFP control PHHs identified 24 genuine C/EBPα target genes that are regulated in response to prolonged VPA exposure in PHHs. Altogether this provides new insights on the involvement of C/EBPα in driving VPA-induced mitochondrial dysfunction in human liver cells. This hub gene, with its downstream regulators involved in this deregulation, thus represent potential new biomarkers for VPA-induced mitochondrial dysfunction.

**Electronic supplementary material:**

The online version of this article (10.1007/s00204-020-02835-x) contains supplementary material, which is available to authorized users.

## Introduction

Valproic acid (VPA) is one of the most widely prescribed drugs for epilepsy. Despite its therapeutic potency, prolonged VPA exposure can induce hepatoxicity which may ultimately progress into more severe liver injury. VPA is a simple fatty acid that is almost entirely metabolized by the liver via several mechanisms, including cytochrome P-450-mediated metabolism, glucuronidation and mitochondrial β-oxidation (Silva et al. 2008). Hallmarks of VPA-induced hepatoxicity include disruption of mitochondrial β-oxidation, reduction of anti-oxidant capacity, and necrosis; factors which may all promote hepatic steatosis.

β-oxidation represents a key mechanism for lipid metabolism, and consequently inhibition of β-oxidation leads to lipid retention in hepatocytes. During β-oxidation of lipids acetylCoenzyme A (CoA) is formed, which enters the tricarboxylic acid (TCA) cycle and is important for ATP generation. The enzyme carnitine palmitoyltransferase 1 (CPT1) plays a key role in this process by shuttling fatty acid-CoA into the mitochondria. CPT1 is affected by the majority of steatohepatitis-inducing drugs. Multiple studies, demonstrate an association between steatosis and mitochondrial dysfunction, which is thought to be mainly due to disrupted β-oxidation (Cengiz et al. 2000; Fromenty et al. 1997; Lheureux and Hantson 2009; Pessayre et al. 1999; Tong et al. 2005).

VPA has been recognized for a long time as a histone deacetylase (HDAC) inhibitor and can thereby affect gene expression and cellular phenotype (Machado-Vieira et al. 2011). Acetylation of histones on lysine residues is controlled by histone acetyltransferases (HATs) and correlates with chromatin remodeling and a permissive chromatin state. Removal of acetylation by HDACs contributes to chromatin compaction and gene silencing. Exposure to VPA can, therefore, exert broad effects on gene expression by preventing global deacetylation of histones and promoting a permissive chromatin state. We have previously shown that prolonged exposure of primary human hepatocytes (PHHs) to VPA can induce gene expression changes as well as changes in 5-methylcytosine DNA methylation patterns (van Breda et al. 2018; Wolters et al. 2017, 2018). Although these studies successfully identify cellular pathways related to mitochondrial functioning, TCA and β-oxidation as primary mechanisms affected by VPA exposure, their relative contribution to VPA-induced steatosis remains unclear.

Biological interpretation of large omics datasets remains difficult (van den Beucken 2019). Distinguishing gene expression changes driving steatosis, from expression changes that are merely bystander effect and thus are not causally related to steatosis, is essential for proper interpretation. We have recently applied a lentiviral shRNA strategy to causally link gene expression profiles to a phenotypic endpoint by knocking-down key TFs (Smit et al. 2017). Prediction of TF activity from transcriptomics data from liver cells exposed to the DNA damaging agent benzo(a)pyrene combined with knock-down of predicted TFs enabled identification of TFs that functionally modified the DNA damage response. This strategy can directly identify causal events driving cellular toxicity mechanisms by reducing complex transcriptome datasets, and can, therefore, be instrumental for biological interpretation of omics datasets.

The aim of the current study was to gain more insights into the adverse effects of repeated VPA exposure by regulating gene expression in PHHs. Based on previously generated gene expression profiles we predicted the activity of all human transcription factors in response to VPA. For one of the top TFs identified, CCAAT/enhancer-binding protein a (C/EBPα), we demonstrated its causal role in mediating mitochondrial dysfunction. In addition, we assessed and curated the C/EBPα-controlled transcriptional network in PHHs using RNA-Seq analysis in C/EBPα knock-down PHHs.

## Materials and methods

### Cell culture

Primary human hepatocytes (PHHs) were cultured as described previously (Wolters et al. 2018). In brief, cryopreserved PHHs (Invitrogen) of three individuals (Hu8119, Hu1591 and Hu1540) were thawed and resuspended according to the supplier’s instructions. Next, PHHs from all individuals were pooled to account for inter-individual variability in toxicant susceptibility and cultured in a double collagen sandwich. The PHHs were maintained in William’s E Medium (WEM) + 20 mL hepatocyte supplement pack (CM4000, Invitrogen) supplemented with 1% penicillin/streptomycin (Gibco) with daily replacement of culture medium. 3 days after seeding, the PHHs were exposed to VPA in the range of 1–30 mM for 1, 2, and 3 day(s) daily or 6.3–50 mM for 3 h. In parallel PHHs were exposed to 1% milli-Q (vehicle control) in culture media for the same duration. We have previously found that 15 mM VPA was required to induce observable steatosis in PHHs (van Breda et al. 2018; Wolters et al. 2017, 2018). Using the same PHH donor pool as used in the current study we previously determined 100%, 96% and 77% cell viability at days 1, 2, and 3, respectively, of daily exposure to 15 mM VPA and 97%, 82% and 44% cell viability at days 1, 2, and 3, respectively, of daily exposure to 30 mM VPA (Wolters et al. 2018).

### Mitochondrial respiration

Mitochondrial respiration was measured via the Agilent Seahorse XF Cell Mito Stress assay according to the manufacturer’s instructions. PHHs were seeded at a density of 4 × 10^4^ cells per well in a collagen sandwich. 36 h post seeding, the PHHs were exposed to 1 mM, 5 mM, 15 mM, and 30 mM of VPA (SIGMA, CAS 1069-66-5) or 1% milli-Q (vehicle control) in culture medium for 1, 2, or 3 day(s) with daily replenishment with freshly prepared VPA or milli-Q bearing culture medium. Upon completion of the treatment the oxygen consumption rate (OCR) was assessed using the Agilent Seahorse XF Extracellular Flux Analyzer (Agilent). A mitochondrial stress test was performed by subsequent injections of 1 μM oligomycin, 0.6 μM FCCP, and 1 μM mixture of rotenone and antimycin A. Thereafter, basal respiration, and maximal respiration, was calculated according to the Seahorse Bioscience guidelines. OCR values were normalized to cell number (well confluency), which was determined after the assay using the IncuCyte Zoom scanner (Essen BioScience, MI, USA). In addition, mitochondrial function was assessed by MTT assay. PHHs were seeded as described above, and 36 h post seeding exposed to 15 mM VPA for 72 h with daily media exchange. During the last 4 h of the exposure 20 μL MTT (5 mg/mL) (Sigma, Zwijndrecht, The Netherlands) was added to all wells. Afterwards, culture medium was removed and acidified DMSO was added to all wells. The absorbance at 540 nm was measured with the iMark Microplate Absorbance Reader (Biorad, Veenendaal, The Netherlands). MTT values were normalized to cell number as described above.

### Prediction of VPA-modulated transcription factors

For the prediction of transcription factor (TF) targets, we used previously generated RNA-Seq data of PHHs (same donor composition as in this study) exposed to 15 mM VPA or vehicle control for 1, 2, 3 days (Wolters et al. 2018). The raw RNA-Seq data are available on arrayExpress (accession number: E-MTAB-5984). TF-target prediction was done using a sign-sensitive methodology as previously described in Essaghir et al. (2010). First, a human TF- target gene network was constructed by retrieving all curated TF-target gene interactions described in MetaCore for 1035 TFs/TF complexes (mined from AnimalTFDB (Zhang et al. 2012)). This collection contained 41,735 interactions targeting 10,409 distinct genes and was used to estimate TF activity in the RNA-Seq data of VPA-exposed PHHs. Differentially expressed genes (VPA-treated versus time-matched controls) were identified using DESeq2 using a Benjamini–Hochberg adjusted *p* value threshold of *p* < 0.05. Next, the human TF-target gene collection was used to estimate TF activity in samples of our RNA-sequencing experiment by employing one-sided Fisher’s exact test (hypergeometric test) on chemical sets, according to the formula: $pval= \sum_{i=k}^{i=n}\left(\genfrac{}{}{0pt}{}{m}{i}\right)\left(\genfrac{}{}{0pt}{}{N-m}{n-i}\right) / \left(\genfrac{}{}{0pt}{}{N}{n}\right)$, where *m* is the number of target genes annotated for the TF, *n* is the number of query genes and *N* the total number of interactions in the TF signature set. *k* varied according to the hypothesis tested, i.e., number of target genes showing (i) same direction or (ii) opposite direction of expression from the annotated signature for the TF tested. TFs were deemed activated/repressed at each time point considering the lowest FDR (Benjamini–Hochberg) after testing both hypotheses; in case a threshold was not reached (FDR < 0.01), the TF was considered not significantly affected.

### RNA interference

Knock-down of C/EBPα was achieved using lentiviral shRNA construct TRCN0000356263, and shRNA construct TRCN0000072179 targeting green fluorescent protein (GFP) as negative control (TRC consortium). Co-transfection of 293 T cells with the indicated shRNA constructs and packaging plasmids pCMVdR8.74psPAX2 and pMD2.G particles was performed to generate lentiviral particles. Lentiviral supernatant was harvested 48 and 72 h post transfection. PHHs were transduced with lentiviral supernatant in PHH cell culture media supplemented with 8 μg/mL polybrene. The following day the viral media was removed and PHHs were cultured for 48 h to allow efficient knock-down prior to the experiment.

### RNA sequencing

PHHs expressing shGFP or shCEBP/α constructs were seeded at 350,000 cells/well in a 24 well plate in a collagen sandwich. Sixteen hours post seeding, PHHs were transduced with lentiviral particles bearing shCEBP/a or shGFP. 48 h post transduction the PHHs were exposed to 15 mM VPA or Milli-Q as vehicle control for 72 h, with daily replenishment of the cell culture medium. Upon exposure PHHs were lysed in 250 μL Trizol/well and RNA was isolated according to the manufacturer's instructions. All RNA samples had a RNA integrity number (RIN) above nine upon quality check using the Agilent 2100 Bioanalyzer RNA 6000 Nano chips (Agilent Technologies). RNA sequence libraries were made utilizing the mRNA-Seq Library Prep Kit V2 (Lexogen) according to the manufacturer’s instructions with 500 ng of RNA as input and 21 cycli to amplify the RNA-Seq library. All samples were pooled at an equimolar concentration of 1.5 nM, and sequenced (5 µL per lane) using an Illumina HiSeq 2000 in 100 bp paired-end reads. Sequence data quality was analyzed by means of FastQC.V0.011.5. Analysis of the RNA-seq data was performed as previously described (Caiment et al. 2015). Briefly, raw reads were trimmed to the first 88 bp with Trimmomatic v0.33 and subsequently aligned to the Ensembl human genome (hg38, release 84) using Bowtie v1.1.1 (Langmead et al. 2009) with the default parameters. Mapping was then quantified by RSEM v1.2.28 (Li and Dewey 2011). Differentially expressed genes were identified using DEseq2 using a Benjamini–Hochberg adjusted *p* value of < 0.05 as threshold.

### Statistics

Student’s *t* test or one-way analysis of variance with Tukey’s post-hoc test was used to test significance between samples. A significance threshold of *p* < 0.05 was applied. Points and error bars plotted in graphs represent the mean ± standard deviation for three or more independent experiments.

## Results

### Prolonged VPA exposure reduces basal respiration rates

**Fig. 1:**
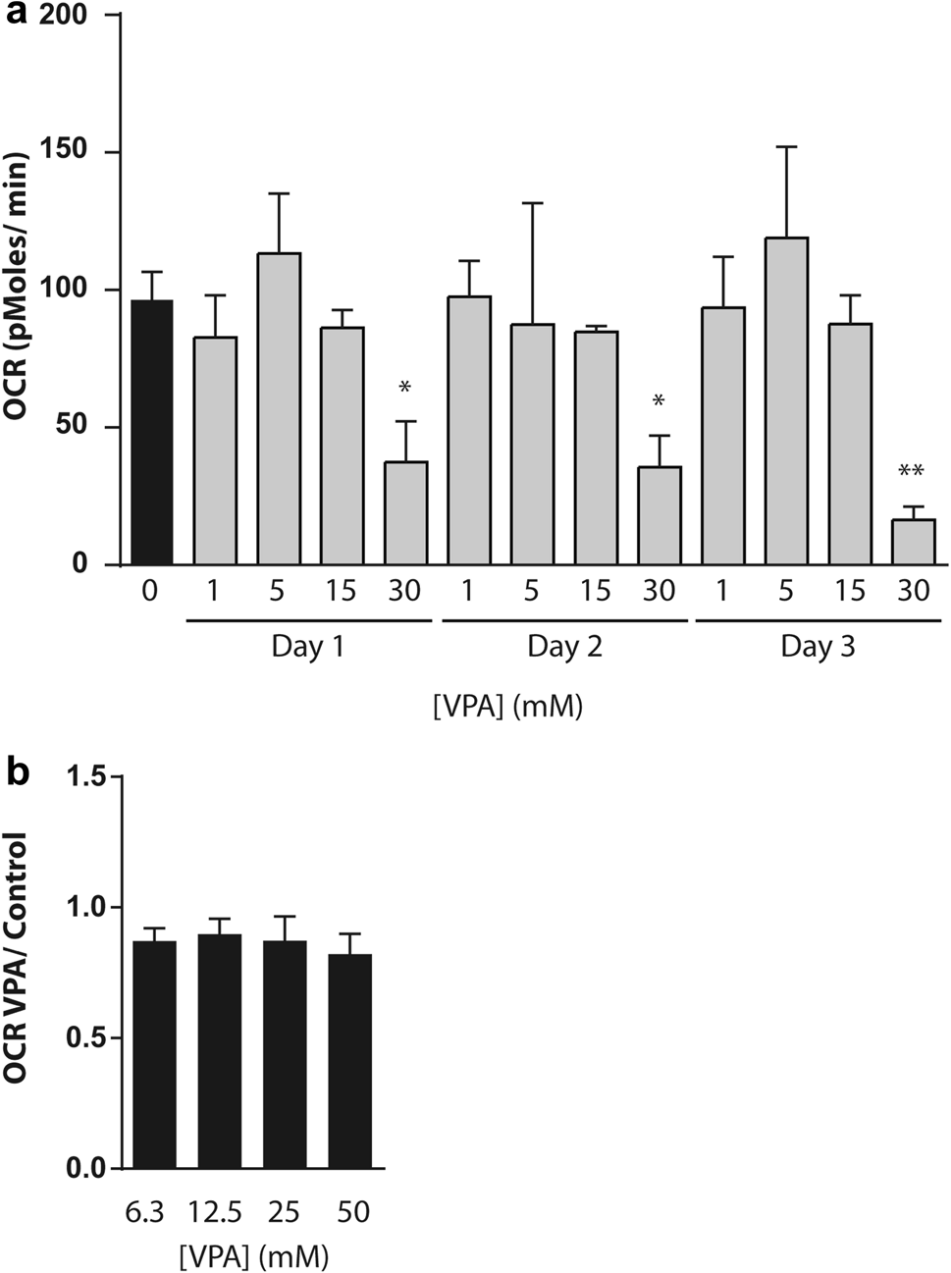
Prolonged daily VPA exposure causes reduced respiration rates in PHHs. **a** PHHs were exposed daily to 1–30 mM VPA or left untreated for 1, 2 or 3 days. Basal respiration rates were subsequently determined using the Seahorse XF extracellular flux analyzer. Average respiration rates are shown relative to vehicle-treated PHHs from three biological replicates. Error bars indicate standard deviation. *p* values obtained with one-way ANOVA, Tukey’s post-hoc test. *p < 0.05, **p < 0.01. **b** PHHs were exposed 6.3, 12.5, 25 or 50 mM VPA or vehicle control for 3 h, after which basal respiration rates were assessed as described in **a**. Average respiration rates are shown relative to vehicle-treated PHHs from three independent biological replicates. Error bars indicate standard deviation

VPA exposure has been associated with mitochondrial dysfunction. Here, we assessed the effect of VPA on oxygen consumption rate (OCR) in PHHs. First, we determined the effect of daily repeated exposure to VPA on basal respiration rates. For this, PHHs were treated with various concentrations of VPA ranging from 1 to 30 mM, for 1, 2 or 3 days. Basal respiration rates were reduced after exposure to 30 mM VPA over 60% on days 1 and 2 (*p* < 0.05) and over 80% on day 3 (*p* < 0.01) compared to vehicle-treated controls (Fig. 1a). This suggests that basal respiration rates drop during prolonged daily VPA exposure. Next, we assessed whether the reduction in basal OCR was already induced by short term exposure to VPA. Exposure of PHHs to increasing amounts of VPA (6.3, 12.5, 25 and 50 mM) for 3 h did not significantly reduce basal respiration rates compared to vehicle control (Fig. 1b). Prolonged exposure (24–72 h) to 30 mM VPA was profoundly more potent in reducing basal respiration compared to 25 or 50 mM VPA for a short period of 3 h. All together these results demonstrate that chronic, daily exposure, to VPA but not a short 3 h exposure, leads to reduced OCR in PHH cells.

### Potential TFs regulated by VPA exposure

**Fig. 2:**
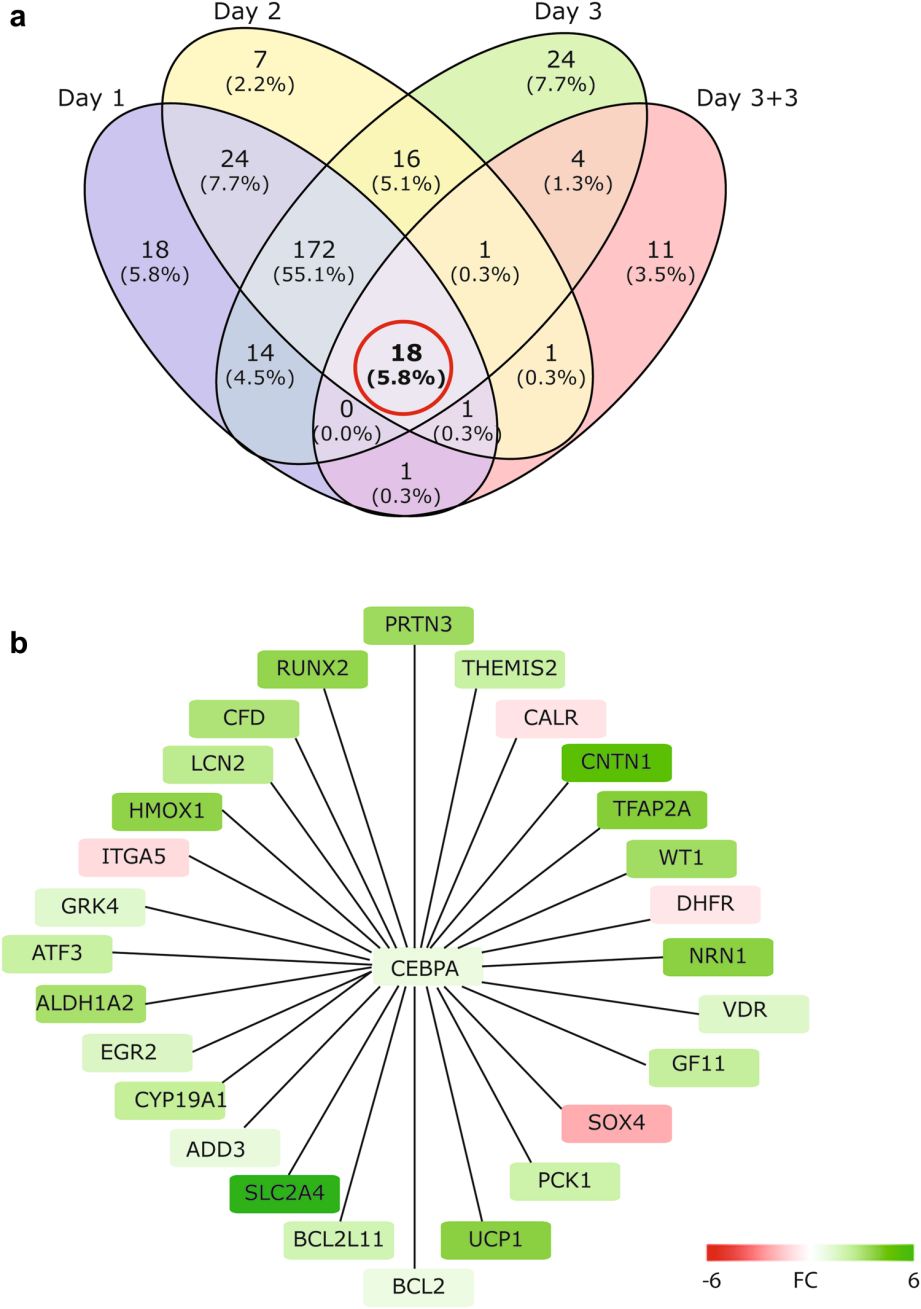
Prediction of TFs underlying VPA-induced transcriptome. **a** VENN diagram depicting all TFs associated with increased gene expression at indicated days of VPA exposure. TFs were identified using DEGs for each day as bait to extract candidate TFs from the MetaCore™ database using a FDR < 0.01. **b** Target gene interaction network surrounding C/EBPα. For the predicted target genes of C/EBPα, the expression changes are shown after 3 days of daily exposure of PHHs to 15 mM VPA using previously published RNA-Seq data

We have previously studied the effect of daily VPA exposure on global gene expression using the same experimental setting and PHH donor pool (Wolters et al. 2018). Here, we used a previously published transcriptome dataset to identify critical TFs that potentially mediate the VPA-induced changes in gene expression, with the underlying idea to subsequently experimentally test the importance of the TF-target gene networks for impairing mitochondrial functioning. Starting point for this analysis was the differentially expressed gene set (DEGs) on days 1, 2 and 3 of VPA exposure as well as DEGs identified after 3 days of exposure followed by a 3 day washout period, using a FDR < 0.05 (Wolters et al. 2018). For each day the DEG list was compared against a curated TF-target gene list comprising 41,735 interactions targeting 10,409 unique genes using a right-sided Fisher’s exact test (FDR < 0.01). TF activity was inferred on the basis of the expression levels of its targets. Overexpression of target genes is indicative for TFs that positively affect transcription based on the curated TF-target gene list. Similarly, under representation of target genes is indicative for TFs that are known to repress gene expression. Using this strategy 248, 240 and 249 TFs were identified that were associated with active gene expression on days 1, 2, and 3, respectively (Fig. 2a). In addition, a 3 day washout period in which the PHHs were no longer exposed to VPA reduced the number of active TFs from 249 to 37. In total 18 TFs were identified that appeared to be activated by prolonged VPA exposure and remained activated after VPA withdrawal (Table 1).

**Table 1:** Predicted TFs underlying persistent gene expression changes upon prolonged VPA exposure

Transcription factor	
AML1/ETO fusion protein	PAX3
BCL6	PGR
CEBPA	POU2F1
CEBPB	PROX1
E2F3	SP1
EBF1	TCF7L1
EOMES	TFAP2A
ETV4	TP63
GFI1	WT1

### Depletion of C/EBPα affects respiration rates in response to daily VPA exposure

**Fig. 3:**
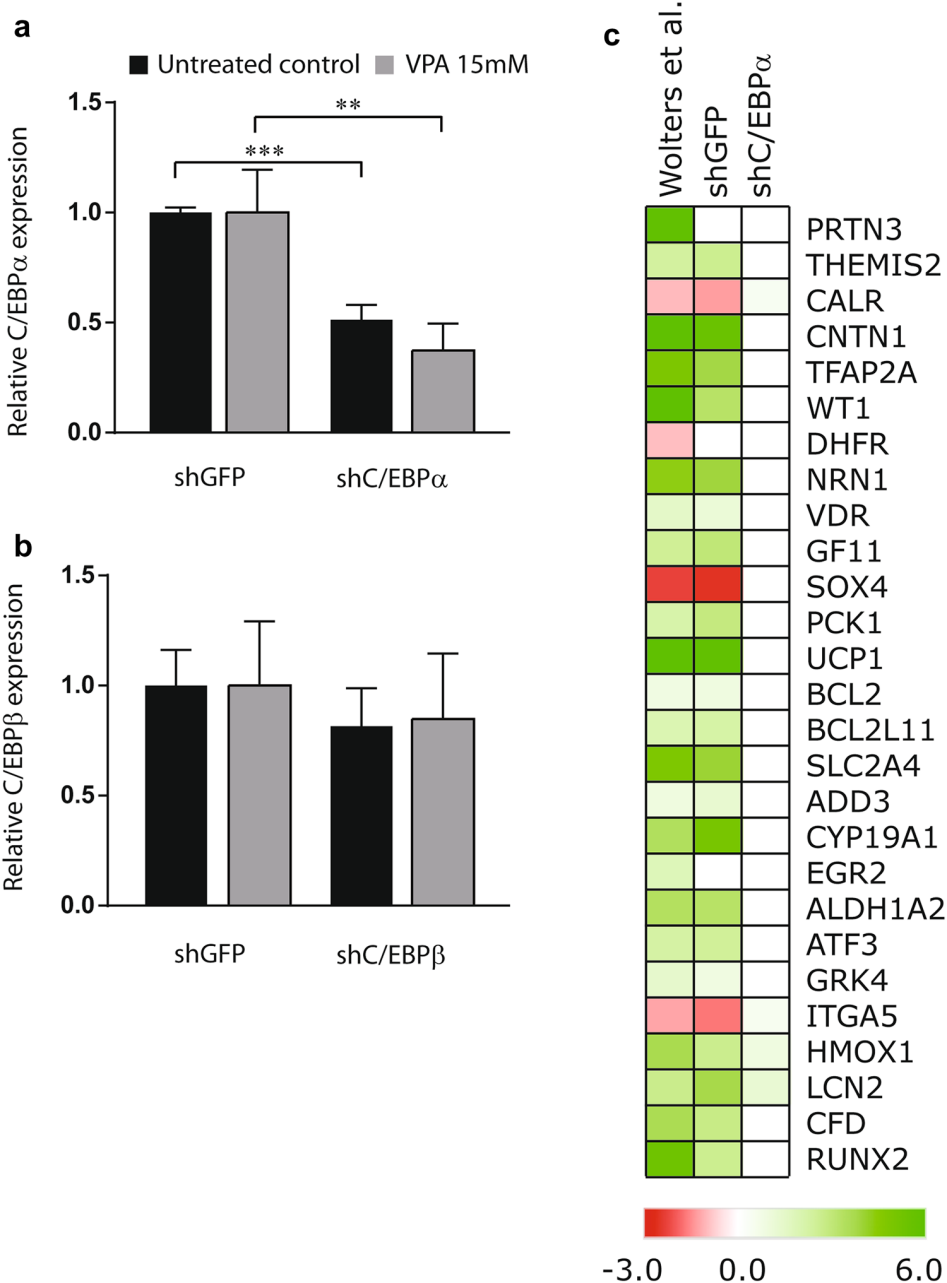
PHHs were transduced with lentiviral shRNA constructs directed at C/EBPα or GFP as control. Subsequently, shC/EBPα and shGFP PHHs were exposed to 15 mM VPA or vehicle control for 3 days. Total RNA was extracted from three independent biological repeats and subjected to mRNA sequencing to identify DEGs using DEseq2 (FDR < 0.05). Relative reads counts normalized to total read number are shown for (**a**) C/EBPα and (**b**) C/EBPβ at 3 days of daily exposure to 15 mM VPA or untreated control. *p* values obtained with one-way ANOVA, Tukey’s post-hoc test. **p < 0.01, ***p < 0.001. **c** Heat map showing the VPA-induced change in expression of the predicted target genes of C/EBPα in previously published dataset (Wolters et al. 2018) (left column). The effect of daily 15 mM VPA exposure for 3 days on the expression of this target gene panel in shGFP (middle column) and shC/EBPα (right column) bearing PHHs is shown

Next, we wanted to improve mechanistic insights into the transcriptional effects of a candidate TF in response to prolonged VPA exposure. The TF C/EBPα was selected for generating proof-of-principle, since it has previously been linked to hepatic steatosis (Guillory et al. 2018). First, we generated the target gene interaction network surrounding C/EBPα based on the DEGs from days 1, 2, 3 and 3 days exposure with 3 days washout that we previously used to identify C/EBPα. This resulting network comprises 27 target genes of which 23 genes are predicted to be positively regulated by C/EBPα in response to VPA and 4 genes were expected to be repressed by C/EBPα in response to VPA (Fig. 2b). TF-target gene interaction networks are cell context-dependent, different subsets of genes can be regulated by the same TF in different cell types (Lambert et al. 2018). Application of a generic catalog of predicted TF-target genes is, therefore, prone to identifying false-positive target genes. For that reason, we assessed whether the C/EBPα target gene interaction network identified here contained false-positive target genes that are not regulated in the cellular context of PHHs. To do this, C/EBPα expression was reduced in PHHs using a lentiviral RNA interference strategy by stably expressing a shRNA targeting C/EBPα. PHHs expressing a shRNA targeting GFP were used as negative control. C/EBPα mRNA expression was reduced twofold in shC/EBPα bearing PHHs compared to shGFP PHHs (*p* < 0.01) (Fig. 3a). Expression of the related gene C/EBPβ was not significantly affected by the shC/EBPα construct (Fig. 3b). PHH-shC/EBPα and shGFP were treated with 15 mM VPA for 72 h after which RNA-Seq was used to generate mRNA expression profiles. We first wanted to determine whether we were able to reproduce the C/EBPα target gene interaction network (Fig. 2b) in this new experiment. For this we analyzed the effect of VPA on the transcriptome of shGFP control cells. Indeed, analysis of the newly generated RNA expression data confirmed VPA-induced regulation for 24 out of 27 of the previously identified target gene network surrounding C/EBPα in the shGFP bearing PHH controls (Fig. 3c). This illustrates the biological reproducibility of the effect of VPA exposure on the expression of these 24 genes. Nevertheless, the regulation of three genes previously identified (PRTN3, DHFR and EGR2) by VPA could not be confirmed in the shGFP bearing PHHs. Next, we determined whether these VPA-induced changes in target gene expression are dependent on C/EBPα by performing the same analysis in shC/EBPα PHHs. Depletion of C/EBPα abolished upregulation of 21 out of 24 target genes upon VPA exposure (Fig. 3c). This indicates that C/EBPα indeed plays a role in regulating their expression in response to VPA. Three genes (CALR, SOX4, and ITGA5) were mildly induced by VPA (~1.4-fold) upon shC/EBPα knock-down, while expression of these genes was repressed by VPA in both the dataset of the previous study, as well as in the shGFP bearing PHHs of the current study. This suggests that C/EBPα acts as a transcriptional repressor on these genes upon VPA exposure and that knock-down of C/EBPα removes this repressive effect. Altogether these data reveal that most predicted transcriptional target genes of C/EBPα are bona fide targets in the context of the PHHs tested here.

### C/EBPα depletion prevents VPA impaired respiration rates

**Fig. 4:**
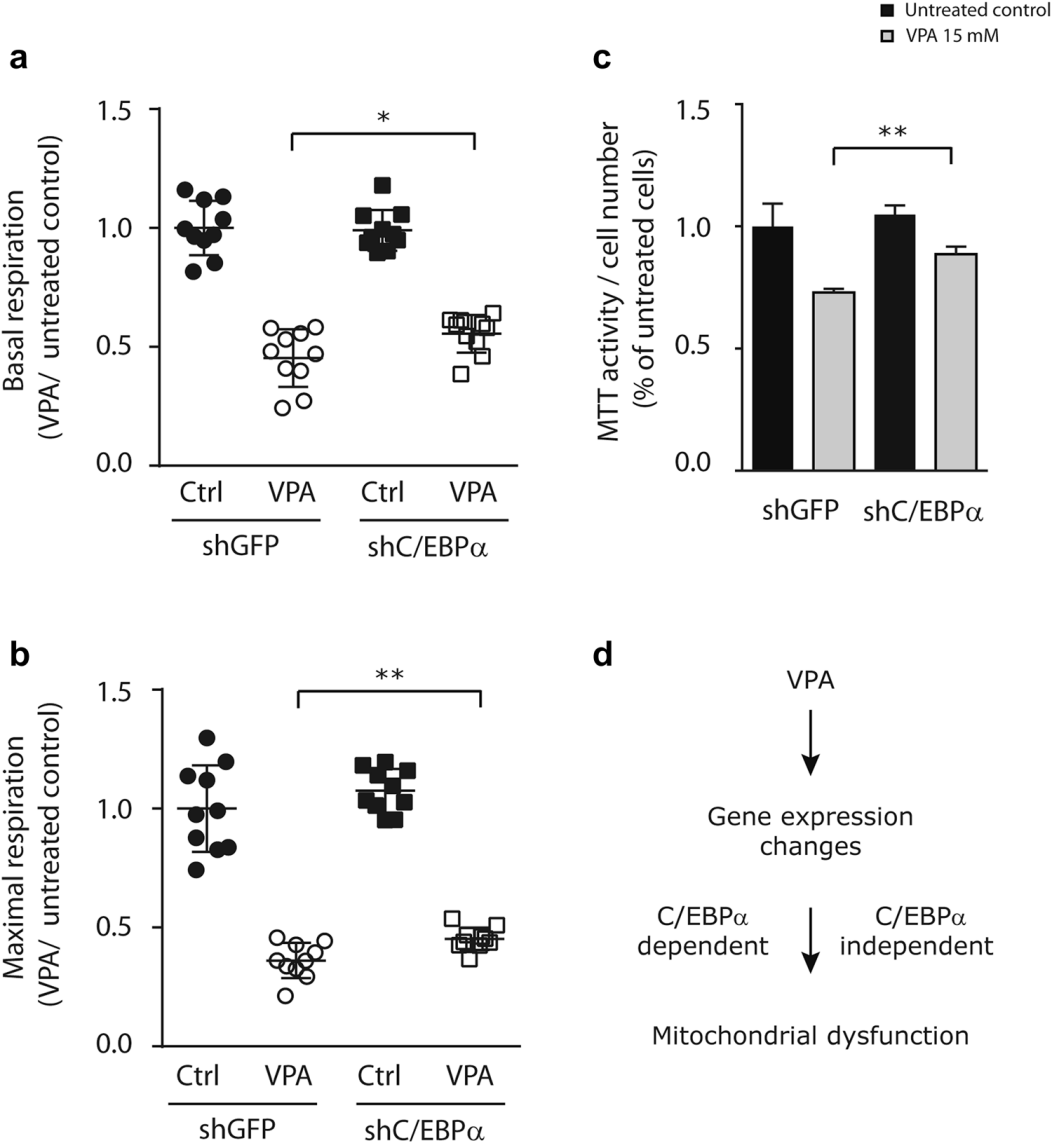
C/EBPα knock-down results in mitochondrial dysfunction. PHHs bearing shC/EBPα or shGFP were exposed daily to 15 mM VPA for 3 days. Subsequently, Seahorse XF extracellular flux analyzer was used to determine (**a**) basal and (**b**) maximal respiration rates. Average respiration rates are shown relative to shGFP PHHs as control. Error bars indicate standard deviation, *p* values obtained with Student’s *t* test *p < 0.05, **p < 0.01. **c** Under the same conditions as in A + B mitochondrial functioning was assessed by MTT assay. Average values are expressed for MTT reduction normalized for cell number and relative to vehicle-treated shGFP control PHHs. Error bars indicate standard deviation, *p* values obtained with Student’s *t* test **p < 0.01. **d** Proposed model for VPA-induced mitochondrial dysfunction

To examine the biological relevance of C/EBPα in regulating VPA-induced mitochondrial dysfunction, we assessed OCR in C/EBPα knock-down and shGFP control PHHs. Similar to our earlier findings, basal and maximal respiration rates were significantly reduced in control PHHs (shGFP) after 3 days of daily treatment with 15 mM VPA compared to vehicle control (Fig. 4a, b). Basal respiration was reduced by 53%, while maximal respiration was reduced by 66% in shGFP-PHHs after VPA exposure for 3 days. In contrast, VPA exposure resulted in only ~ 45% basal and ~57% maximal respiration in shC/EBPα-PHHs (Fig. 4a, b). Knock-down of C/EBPα resulted in significantly higher basal (*p* < 0.05) and maximal respiration (*p* < 0.01) rates compared to shGFP control PHHs. These results demonstrate that C/EBPα is partially responsible for the reduction in basal and maximal respiration in PHH upon repeated VPA exposure.

To confirm these results we assessed the consequences of C/EBPα knock-down on mitochondrial activity after prolonged VPA exposure by MTT assay. In line with our previous results, we observed a ~30% reduction in MTT activity in shGFP control PHHs after VPA exposure (Fig. 4c). In contrast, the shC/EBPα PHHs only demonstrated a ~12% reduction in response to VPA. Therefore, shC/EBPα PHHs maintained higher mitochondrial activity compared to shGFP control PHHs upon prolonged VPA exposure (*p* < 0.01). All together these results illustrate that C/EBPα plays a causal role in regulating mitochondrial functioning in response to prolonged VPA exposure.

## Discussion

VPA is one of most frequently prescribed anti-epileptic drugs and it is well known that VPA can cause liver injury. Still, the mechanisms underlying VPA-induced liver injury remain poorly understood. In this study we combined a transcriptomics approach with functional genomics to analyze the molecular basis of VPA-induced liver injury. Our data suggests that the transcription factor C/EBPα orchestrates gene expression changes upon prolonged VPA treatment (3 days) that contribute to mitochondrial dysfunction. These findings establish that prolonged, but not a short (3 h) VPA exposure contributes to liver injury partly via regulating C/EBPα-dependent gene expression.

Mitochondrial dysfunction plays a significant role in the development of hepatosteatosis. Our results demonstrate that repeated dose exposure to VPA triggers defects in mitochondrial functioning in PHHs. Up to 53% reduction in basal respiration rate and 66% in maximal respiration rate were robustly found upon repeated VPA dosing for 72 h. This is to our knowledge the first time that reduced OCR in response to repeated VPA exposure has been demonstrated in PHHs. Similar findings have been reported using the liver cancer cell line HepG2 (Komulainen et al. 2015). In addition, VPA has been shown to cause reduced mitochondrial membrane potential in HepG2 cells (Wang et al. 2011) and in rat hepatocytes (Pourahmad et al. 2012; Tong et al. 2005). Interestingly, short term exposure had no effect of oxygen consumption in our study. The absence of a short term VPA effect on OCR was also reported using mitochondria isolated from hepatocytes of mice treated with VPA for 4 h (Torres et al. 2019).

The observation that prolonged VPA exposure is required for mitochondrial dysfunction suggests that this, at least to some degree, depends on gene expression changes. Several studies have shown that daily repeated exposure to VPA for extended periods of time can induce profound changes in gene expression. This includes work done using PHH (Igarashi et al. 2014; van Breda et al. 2018; Wolters et al. 2018) as well as human dermal stem cell‑derived hepatic cells (Rodrigues et al. 2016), primary rat hepatocytes (De Abrew et al. 2015) and in vivo exposed mouse (Vitins et al. 2014) or rat hepatocytes (Igarashi et al. 2014).

While these studies identify a pleiotropy of candidate genes including known steatotic markers that might play a role in VPA-induced adverse outcomes, the biological interpretation and identification of causal interactions from these data as such remains difficult. Here we reduced the complex gene expression profiles triggered by VPA into more comprehensible panels of TFs. We previously applied this approach successfully for causally linking benzo[a]pyrene-induced gene expression profiles to the presence of DNA damage (Smit et al. 2017). In the current study, ~250 TFs were identified that could explain the expression changes observed during prolonged VPA exposure. The number of potentially involved TFs was strongly reduced after a 3 day washout period. This illustrates that most gene expression changes return to normal and are thus reversible upon VPA withdrawal. Similar finding were previously found in our laboratory (van Breda et al. 2018). Eighteen TFs were identified that correlated with persistent gene expression changes and thus might be of particular interest to study long-term adverse effects of VPA.

The TF C/EBPα was selected for further study as it has previously been linked to hepatic steatosis (Guillory et al. 2018). Homozygous deletion of CEBPA is postnatally lethal due to hypoglycemia (Wang et al. 1995), whereas liver-specific conditional knockout of CEBPA results in glucose intolerance and steatosis (Matsusue et al. 2004). Our data shows that knock-down of C/EBPα in PHHs partially prevents reduced OCR upon prolonged VPA exposure. We observed a significant, but incomplete rescue from the VPA-induced mitochondrial dysfunction assessed by OCR and MTT measurements. This suggests that C/EBPα, and thus likely, target genes under control of C/EBPα, are causally involved in the molecular mechanism affected by VPA leading to impaired mitochondrial function (Fig. 4d). The relatively small rescue effect of C/EBPα knock-down on mitochondrial function can be interpreted in multiple ways. First, the knock-down of C/EBPα was only ~50% compared to control cells. Although this reduction in C/EBPα expression was sufficient to prevent expression of C/EBPα target genes and to affect mitochondrial function, it may be possible that further depletion of C/EBPα levels would have a larger impact on mitochondrial function. Second, transcriptional changes driven by other TFs, are likely to affect mitochondrial dysfunction upon prolonged VPA exposure. Third and last, mitochondrial dysfunction might be independent of dynamic transcriptional regulation (Fig. 4d). Lentiviral shRNA or CRISPR/Cas9 screening of all human TFs could be performed to identify additional TFs that modulate mitochondrial function in response to VPA (Rancati et al. 2018).

Here, we successfully confirmed the effect of VPA on expression of C/EBPα-dependent genes that we identified in a transcriptome dataset from a previous study (Wolters et al. 2018). Out of 27 predicted C/EBPα target genes, 24 were experimentally confirmed as C/EBPα-dependent, by performing RNA-Seq analysis in VPA-treated C/EBPα knock-down PHHs. Three genes, PRTN3, DHFR and EGR2 could not be confirmed as VPA-regulated C/EBPα target genes in PHHs. One explanation for this could be that although PRTN3, DHFR and EGR2 might be putative transcriptional targets of C/EBPα, in the cellular context of PHHs in this study, they may not be. Possibly the chromatin state of these loci may not be permissive for active gene expression. This illustrates the difficulty of inferring TF activity from gene expression profiles. The current catalog of human TF- target gene interactions is largely built on the presence of TF binding consensus sites present within the promoter region of putative target genes. It has been reported that for over the 1600 likely human TFs a consensus sequence is known for only two-thirds of them (Lambert et al. 2018). Perhaps more importantly, the same TF is capable of controlling expression levels of distinct genes in different cell types (Gertz et al. 2012). Cell type specificity can be obtained by performing chromatin immunoprecipitation sequencing (ChIP-seq) experiments. The ENCODE consortium has generated hundreds of ChIP-seq datasets in various cellular backgrounds (Dunham et al. 2012); however, inferring TF binding sites from these data remains problematic (Furey 2012). Ultimately, experimental validation of the human TF-target gene interaction catalog is vital for adequate interpretation of the many transcriptome datasets that are generated at high pace within the domain of biomedical sciences. In addition, it will be important to understand the relative contribution of the C/EBPα-driven genes in promoting mitochondrial dysfunction after repeated, daily VPA exposure. Several of the C/EBPα-dependent genes that we have identified in PHHs have previously been linked to mitochondrial dysfunction, including uncoupling protein 1 (UCP1) (Demine et al. 2019), heme oxygenase 1 (HMOX1) (Cheng et al. 2009), and activating transcription factor 3 (ATF3) (Kim et al. 2017). This is in line with our observations that C/EBPα partly drives mitochondrial dysfunction. It remains to be determined whether these genes work in concert as part of a larger network or whether any of these genes can impact independently on mitochondrial function. In addition, the role of the other C/EBPα-dependent genes not previously connected to mitochondrial dysfunction remains unclear. To elucidate this, knock-down models for these 24 genes would need to be developed followed by experimental validation of the effect of repeated, daily VPA exposure on mitochondrial dysfunction.

Exploring the initial trigger will be of major interest for understanding the adverse effects of VPA. However, the molecular mechanisms responsible for C/EBPα activation by VPA are unclear. VPA has been shown to affect multiple signaling pathways. MAPK/ERK signaling can be activated by VPA, while inhibitory effects have been reported on GSK3β, Insp3, PKC and class I HDAC (Friedman 2015). We hypothesize that the VPA-induced gene expression changes that are C/EBPα dependent are driven by phosphorylation of C/EBPα. It would be interesting to assess the phosphorylation status of C/EBPα in response to prolonged VPA exposure. It has been reported that C/EBPα harbors eight potential phosphorylation sites (Tsukada et al. 2011). For example ERK has been shown to phosphorylate C/EBPα at Ser 21 (Ross et al. 2004), while C/EBPα is phosphorylated by PI3K/Akt at Ser 193 (Wang et al. 2004), by GSK3β at T222 and T226 (Ross et al. 1999) and by Ras as acts on Ser 248 (Behre et al. 2002). Hence, the upstream kinases responsible for C/EBPα activation may be inferred based on the phosphorylation pattern of C/EBPα, thereby providing valuable information of the signaling events affected by prolonged VPA exposure that could promote mitochondrial dysfunction.

In conclusion, this study illustrates that prolonged VPA exposure promotes mitochondrial dysfunction in PHHs. Mitochondrial dysfunction appears at least in part, driven by C/EBPα-dependent gene expression. Identification of causally related signaling events upstream of C/EBPα and downstream target genes will help to better understand the molecular basis of VPA-induced mitochondrial dysfunction. This work should be extended to other TF gene networks beyond C/EBPα to obtain a more comprehensive picture of the VPA-induced effects in PHHs. The strategy applied here offers great opportunities for future studies to evaluate causal relationships between compound-induced gene expression changes and phenotypic endpoints. This approach can be used to exploit the enormous amounts of publically available big data within the toxicology domain and can contribute to the biological interpretation of such complex datasets.

## Electronic supplementary material

Below is the link to the electronic supplementary material.

Supplementary file1 (TIF 29,853 kb)
