# Supplementary figures and images for: Valproic acid promotes mitochondrial dysfunction in primary human hepatocytes in vitro; impact of C/EBPα-controlled gene expression
Source: Arch Toxicol. 2020 Jul 4;94(10):3463–73. doi: 10.1007/s00204-020-02835-x (PMC7502062; doi:10.1007/s00204-020-02835-x)

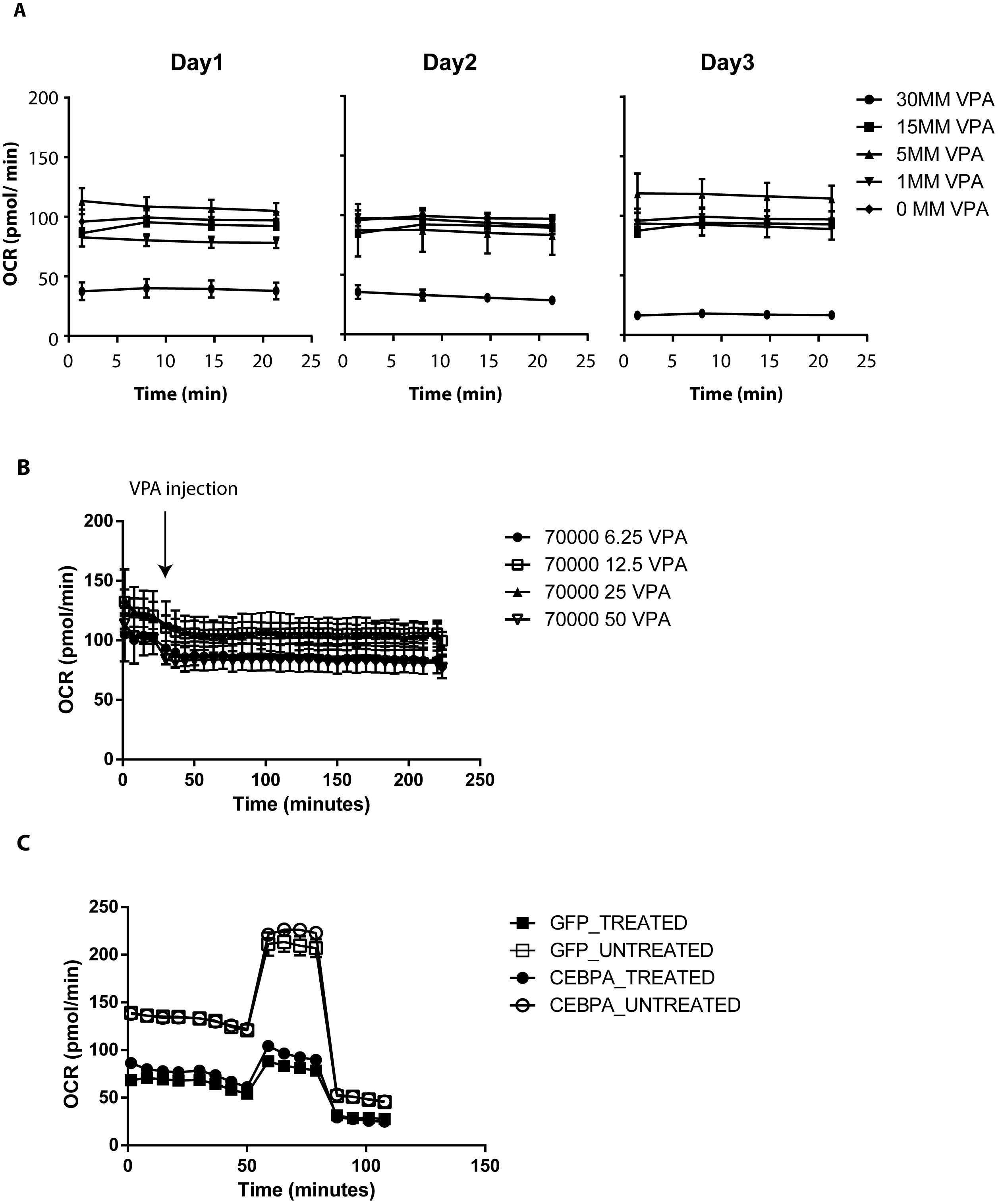

Supplement: Supplementary file 1 — Supplementary file1 (TIF 29,853 kb) [file 204_2020_2835_MOESM1_ESM.tif]
